# Assessing the Relative Impact of Diverse Stressors among Public Safety Personnel

**DOI:** 10.3390/ijerph17041234

**Published:** 2020-02-14

**Authors:** R. Nicholas Carleton, Tracie O. Afifi, Tamara Taillieu, Sarah Turner, Julia E. Mason, Rosemary Ricciardelli, Donald R. McCreary, Adam D. Vaughan, Gregory S. Anderson, Rachel L. Krakauer, Elizabeth A. Donnelly, Ronald D. Camp, Dianne Groll, Heidi A. Cramm, Renée S. MacPhee, Curt T. Griffiths

**Affiliations:** 1 Department of Psychology, Anxiety and Illness Behaviours Laboratory, University of Regina, Regina, SK S4S 0A2, Canadarachellkrakauer@gmail.com (R.L.K.); 2 Rady Faculty of Health Sciences, University of Manitoba, Winnipeg, MB R3E 0W3, Canada; Tracie.Afifi@umanitoba.ca (T.O.A.); Tamara.Taillieu@umanitoba.ca (T.T.); Sarah.Turner@umanitoba.ca (S.T.); 3 Department of Sociology, Memorial University of Newfoundland, Saint John’s, NL A1C 5S7, Canada; rricciardell@mun.ca; 4 Donald McCreary Scientific Consulting, Vancouver Island, BC V9K 2R8, Canada; donmccreary@yahoo.ca; 5 Office of Applied Research and Graduate Studies, Justice Institute of British Columbia, New Westminster, BC V3L 5T4, Canada; avaughan@jibc.ca (A.D.V.); ganderson@jibc.ca (G.S.A.); 6 School of Social Work, University of Windsor, Windsor, ON N9A 0C5, Canada; donnelly@uwindsor.ca; 7 Hill-Levene Schools of Business, University of Regina, Regina, SK S4S 0A2, Canada; Ronald.Camp@uregina.ca; 8 Departments of Psychiatry and Psychology, Queen’s University, Kingston, ON K7L 3N6, Canada; grolld@queensu.ca (D.G.); heidi.cramm@queensu.ca (H.A.C.); 9 Department of Kinesiology and Physical Education, Wilfrid Laurier University, Waterloo, ON N2L 3C5, Canada; rmacphee@wlu.ca; 10 School of Criminology, Simon Fraser University, Burnaby, BC V5A 1S6, Canada; griffith@sfu.ca

**Keywords:** public safety personnel, potentially psychologically traumatic events, occupational stress, organizational stress, operational stress, mental health disorders

## Abstract

Public Safety Personnel (PSP; e.g., correctional workers and officers, firefighters, paramedics, police officers, and public safety communications officials (e.g., call center operators/dispatchers)) are regularly exposed to potentially psychologically traumatic events (PPTEs). PSP also experience other occupational stressors, including organizational (e.g., staff shortages, inconsistent leadership styles) and operational elements (e.g., shift work, public scrutiny). The current research quantified occupational stressors across PSP categories and assessed for relationships with PPTEs and mental health disorders (e.g., anxiety, depression). The participants were 4820 PSP (31.7% women) responding to established self-report measures for PPTEs, occupational stressors, and mental disorder symptoms. PPTEs and occupational stressors were associated with mental health disorder symptoms (*p*s < 0.001). PSP reported substantial difficulties with occupational stressors associated with mental health disorder symptoms, even after accounting for diverse PPTE exposures. PPTEs may be inevitable for PSP and are related to mental health; however, leadership style, organizational engagement, stigma, sleep, and social environment are modifiable variables that appear significantly related to mental health.

## 1. Introduction

Public safety personnel (PSP; e.g., correctional workers and officers, firefighters, paramedics, police officers, and public safety communications officials (e.g., call center operators/dispatchers)) form a diverse set of vocations focused on protecting our populations from threat and harm [1]. As a function of their occupations, PSP are frequently exposed to a wide variety of workplace stressors. Most research examining these occupational concerns has focused on the exposure to potentially psychologically traumatic events (PPTEs; e.g., threatened or actual physical assaults, sexual violence, fires, and explosions; [1,2,3]). That is, persons working in public safety tend to experience PPTEs at much higher frequencies than the general public [3,4,5]. Research has also demonstrated that PPTE exposure is associated with an increased risk of negative mental health outcomes, such as symptoms of posttraumatic stress disorder (PTSD; [2]), major depressive disorder (MDD; [6]) panic disorder (PD; [7]), generalized anxiety disorder (GAD; [8]), social anxiety disorder (SAD; [9]), and alcohol use disorder (AUD; [10]). Results from a recent survey with a large Canadian PSP sample indicated that the lifetime average number of different PPTE types experienced was 11.08 out of 16 different assessed PPTEs, with each type having been experienced 11 or more times by up to 71.3% of respondents [11]. Relatedly, approximately 44.5% of participants in the same study screened positive for one or more mental health disorders [12]. The apparent elevated mental health risk for PSP workers has led to an increased interest in better understanding the associations between workplace stressors and mental health outcomes in this community [1].

There is increasing awareness that PPTEs may not be the only work-related stressor PSP experience. Previous research has suggested that PSP report experiencing a wide range of both general and occupationally-specific stressors [13]. For example, two recently published qualitative studies of PSP implied that PPTE exposures are only one element impacting their mental health [14,15]. Both studies implicated a range of occupational challenges, including issues such as differential treatment of employees by leadership, indifference to mental health, insufficient recognition of stressors, overt and covert stigma, and systemic economic pressures to perpetually do more with less.

Research into the general aspects of workplace stress has typically used theoretical models from occupational health psychology, such as the Job Demands-Control and the Job Demand-Control-Support variation [16,17], Job Demand-Resources [18], and Organizational Justice [19,20] models. The concepts underlying such models emphasize a series of common elements across most jobs, which can lead to workplace stress and individual strain. Concerns have been raised that work–strain models, such as these, have been tested in overly homogenous job contexts [21]; nevertheless, these models have been successfully used in several PSP contexts [22,23,24].

Studies within various PSP occupations, however, suggests that many of the occupational stressors associated with high job demands, low levels of resources, control, and social support, as well as perceptions of poor organizational justice, can be seen as falling into two higher order constructs: organizational and operational stressors [13,25]. Organizational stressors are frequently defined as the stressors associated with job context or setting. Examples of organizational stressors include staff shortages, a lack of training on new equipment, a lack of appropriate resources, inconsistent leadership styles, and a perceived lack of support between co-workers and leaders [13,14,26,27]. Organizational management structure, as well as the resulting policies and practices, can also become sources of daily occupational stress [28]. Operational stressors, on the other hand, typically refer to the stressors directly tied to work content or duties. Examples of operational stressors can include PPTEs, but also issues such as fatigue from shift work and overtime, job-related risk of injury (e.g., lower back pain), social life limitations, and the inescapability of work [13,14,26]. Previous research has supported the use of the operational and organizational constructs in a wide variety of PSP occupations, including police [13,28,29], firefighters [30], paramedics [31], and corrections [32].

The costs of occupational stress to individuals (e.g., the increased risk of negative physical and mental health outcomes) and organizations (e.g., absenteeism, employee turnover, poor quality control, and frequent and severe accidents) have been well documented [33,34]. However, research on the effects of operational and organizational stressors on PSP mental health remains nascent. Research is particularly limited regarding the unique impact of PPTEs, operational stressors, and organizational stressors, and whether occupational stressors and PPTEs interact to adversely influence PSP mental health. For example, with regard to the issue of the PPTE and occupational stress interaction, the following question can be asked: are PSP who have experienced more PPTEs and have also experienced higher levels of occupational stress, more likely to have poorer mental health outcomes? To date, we are unaware of any research that has addressed such issues.

Questions regarding the impact of PPTEs relative to operational and organizational stressors on PSP are important because PSP work necessarily involves increased exposure to PPTEs [1,11]; as such, reducing PPTE exposure for PSP, so as to limit the risk of adverse mental health outcomes, may not be a realistic goal. Accordingly, researchers, managers, and policy makers may want to shift their focus to the potential benefits of modifying specific operational and organizational stressors in order to protect PSP mental health. Justifying such workplace modifications would require evidence that PSP-specific occupational stressors (e.g., finding time to stay in good physical condition; fatigue; making friends outside the job; working alone at night; overtime demands; inconsistent leadership styles) are uniquely associated not only with negative mental health in general [35] but especially after controlling for PPTE exposures. There is initial research from a large sample of emergency medical services (EMS) workers indicating that such a relationship exists [31]. Specifically, Donnelly’s study evidenced that organizational stressors, operational stressors, and PPTEs were each significantly and uniquely associated with symptoms of posttraumatic stress. Controlling demographic factors and critical incident stress (i.e., PPTEs), organizational and operational stress retained a significant predictive relationship to posttraumatic stress. Additionally, operational stress was found to interact with critical incident stress (i.e., PPTEs) and alcohol use to further influence the risk of posttraumatic stress. The results were partially replicated in a smaller sample in the Canadian context, where operational stress, both independently and interacting with critical incident stress, was a significant predictor of posttraumatic stress [36]. The results were promising but were focused on paramedicine. If occupational stressors can be demonstrated as having unique relationships with mental health symptoms after partialling out the associations of PPTEs within diverse PSP professionals across a diverse set of mental disorder symptoms, then there would be sufficient support for efforts to modify specific organizational and operational stressors. Importantly, demonstrating significant associations for overall mean levels of organizational and operational stressors alone would not provide sufficient justification or detail for managers and policy makers to invest in significant and specific changes; instead, managers and policy makers need specific details regarding which workplace stressors are most strongly associated with poor mental health functioning. As such, researchers need to extend prior research [13,31,37] by focusing on both overall mean and item-level associations so that the latter can be targeted for modification (e.g., leadership training, wellness interventions, peer support, structural changes to work, limits to shift/over time demands).

The current research was designed to address five main research questions. The initial two questions were examined across PSP categories; however, because the remaining questions were focused on mental health caseness (so as provide easily accessible information for PSP leaders and managers), we were not able to test for PSP differences in our last three research questions. For our first research question, we assessed for differences in overall organizational and operational stressors across PSP categories. Second, we assessed for item-level differences in organizational and operational stressors across PSP categories. For both questions, we hypothesized that there would be variation across PSP categories but had no specific hypotheses about which categories would be higher than others. Third, we assessed for associations between positive screens on several mental health outcomes (i.e., PTSD, MDD, GAD, SAD, PD, AUD) and the overall scores for operational and organizational stress, as well as separately for each occupational stressor. We hypothesized that there would be significant and positive associations between our stress measures/items and screening positive for a mental health condition. Fourth, we assessed for unique associations between overall operational and organizational workplace stress scores and a positive screen on several mental health outcomes, after controlling for PPTE exposure types. We hypothesized that, after controlling for PPTE exposure, there still would be significant associations between both organizational and operational stress scores and positive screens for mental disorders. Fifth, we assessed the extent to which the interaction between trauma exposure and occupational stress predicted a positive screen for mental health. We hypothesized that there would be a significant interaction, such as those with high levels of both PPTEs and occupational stress would be more likely to screen positive for mental health disorders.

### Highlights

Public safety personnel reported substantial difficulties with occupational stressors (e.g., staff shortages, inconsistent leadership styles; shift work, public scrutiny). Occupational stressors were significantly associated with several anxiety and mood related disorders, even after controlling for potentially psychologically traumatic events. Modifying leadership style, organizational engagement, stigma, and social environments for public safety personnel may help mitigate mental health disorders such as posttraumatic stress disorder.

## 2. Materials and Methods

### 2.1. Data and Sample

An online self-report survey was used, following established guidelines for web-based data collection [38]. The survey was made available to PSP participants in English or French as part of a larger study assessing the prevalence of mental disorders among PSP (see [12]). Participants were recruited through emails sent to actively-working PSP, including civilian members working for police and volunteer firefighters. A total of *n* = 8520 PSP responded to the first question of the survey (i.e., “Please indicate which category of First Responders or other Public Safety Personnel you feel best describes your current occupation”), but only *n* = 4820 PSP provided enough information to be definitively placed into one of the six PSP categories of interest in our study (i.e., correctional workers and officers, federal police (i.e., Royal Canadian Mounted Police; (RCMP)), firefighters, paramedics, municipal/provincial police, public safety communications officials (e.g., call center operators/dispatchers)) and proceeded far enough in the survey to complete the occupational stressors and mental disorder sections (56.6% of the total sample). An additional 379 inconsistent respondents were flagged and excluded from the final data analysis because the participant did not respond appropriately to a control question embedded in the trauma and stressors module of the survey. Our final sample consisted of *n* = 4441 PSP (52.1% of the total sample). Ethics approval was obtained from the first author’s University Institutional Research Ethics Board (File #2016-107). Researchers interested in reviewing the data to independently reassess the current results can contact the corresponding author.

### 2.2. Measures

**Occupational Stressors.** Occupational stressors were assessed with the 20-item Organizational Police Stress Questionnaire (PSQ-Org) and the 20-item Operational Police Stress Questionnaire (PSQ-Op; [13]). The PSQ-Org assesses stressors associated with the organization and culture within which the job is performed, including the impact of work on family and social life (e.g., fatigue, occupation-related health issues, not enough time to spend with friends and family); in contrast, the PSQ-Op assesses stressors associated with doing the job (e.g., dealing with co-workers, staff shortages, inconsistent leadership, see Table 1). Despite the scale titles, none of the items are specific to policing, such that each item can apply to other PSP professionals; indeed, the scales have been used successfully with a wide range of PSP [28,29,30,31,32]. Each item on both the PSQ-Org and PSQ-Op is rated on a 7-point Likert scale ranging from 1 (*no stress at all*) to 7 (*a lot of stress*). The overall mean scores on the PSQ-Org and PSQ-Op were computed separately by summing responses across all of the items and dividing by 20, as per the measures’ instructions (McCreary and Thompson, 2006). Individual item means were also computed for each individual PSQ-Org and PSQ-Op item. Cronbach’s alpha for the overall mean score on the PSQ-Org was 0.94 and on the PSQ-Op it was also 0.94, indicating acceptable internal consistency for each measure in the current study.

**Total Number of PPTE Types.** The Life Events Checklist for the Diagnostic and Statistical Manual of Mental Disorders, 5th edition (LEC-5; [39,40]) was used to assess participants’ lifetime exposure to 16 PPTE types (i.e., life threatening natural disaster; fire or explosion; serious transportation accident; serious accident at work, home, or during recreational activity; exposure to a toxic substance; physical assault; assault with a weapon; sexual assault; other unwanted or uncomfortable sexual experience; combat; captivity; life threatening illness or injury; severe human suffering; sudden violent death; sudden accidental death; serious injury, harm, or death you caused to someone else). Participants were also able to provide a response to an “any other incident” category; however, there were too many missing values in the “any other incident” category to defensibly include the category in the current analyses, which meant that the associated responses were not analyzed here. Two LEC-5 items were modified for PSP: specifically, “natural disaster” was revised to “a life-threatening natural disaster” and “transportation accident” was revised to “a serious transportation accident”. The total number of PPTE types was computed by summing exposures across the 16 item types. Participant responses were coded as having been exposed to a specific PPTE if they reported that: (a) it happened to them personally, (b) they witnessed it happen to someone else, (c) they learned about it happening to a close family member or close friend, and/or (d) they were exposed as part of their PSP work. The percentage of missing responses on each individual item was small (ranged from 1.4% to 11.0%), but the cumulative missing values compromised computation of the exact number of PPTE for several participants. Therefore, up to two missing values were allowed in the calculation of the total number of PPTE types. The result excluded another 761 respondents with 3 or more missing values from the analyses where the total number of PPTE types were being considered. The mean number of PPTE types across all exposure frames was 11.08 (*SD* = 3.23) in this sample.

**Mental Disorder Symptom Screens.** Current mental disorder symptoms were assessed using several reliable, validated self-report mental disorder screening measures. PTSD symptoms over the past month were assessed with the PTSD Check List 5 (PCL-5) [39,40,41,42,43]. A positive screen for PTSD was indicated if the participant reported at least one PPTE exposure on the LEC-5 (PTSD follow-up questions were based on single worst traumatic event, most distressing event, or the event that was currently causing the most distress; i.e., Criterion A), met the minimum criteria on each PTSD cluster, and had a total score >32 on the PCL-5 [40]. Depressive symptoms over the past two weeks were assessed with the 9-item Patient Health Questionnaire (PHQ-9) [44,45,46]. A positive screen for MDD was indicated by a total score > 9 on the PHQ-9 [47]. GAD symptoms over the past two weeks were assessed with the 7-item Generalized Anxiety Disorder scale (GAD-7; [48,49,50]). A positive screen for GAD was indicated by a total score >9 on the GAD-7 [51]. Current symptoms of SAD were assessed with the 14-item Social Interaction Phobias scale (SIPS) [52,53,54,55,56]. A positive screen for SAD was indicated by a total score >20 on the SIPS [52]. PD symptoms over the past week were assessed with the 7-item Panic Disorder Symptoms Severity scale (PDSS) based on a past 7-day timeframe [57,58,59]. A positive screen for PD was indicated by a total score >7 on the PDSS [58]. AUD symptoms over the past 12 months were assessed with the Alcohol Use Disorders Identification Test (AUDIT) [60,61]. A positive screen for AUD was indicated by a total score >15 on the AUDIT [60]. Participants were also asked whether they had ever been diagnosed with several other mental disorders including obsessive-compulsive disorder, persistent depressive disorder, bipolar I, bipolar II, and cyclothymic disorder. The low self-reported prevalence of these disorders in this sample precluded the examination of each specific self-reported mental disorder with PPTEs or occupational stressors. A dichotomous positive mental disorder screen was computed based on whether the participant had a positive screen on one or more screening measures and/or self-reported a mental disorder diagnosis.

**Sociodemographic Covariates.** Sociodemographic covariates included sex (i.e., male or female), age (i.e., 19 to 29 years, 30 to 39 years, 40 to 49 years, 50 to 59 years, or 60 years and older), marital status (i.e., married/common-law, remarried, separated/divorced/widowed, or single), race/ethnicity (i.e., white or visible minority), education (i.e., high school or less, some post-secondary less than 4-year college/university program, or university degree/4-year college or higher), urban versus rural work location (i.e., urban or rural), region of residence (i.e., Western Canada, Eastern Canada, Atlantic Canada, Norther Territories), and total years of service (i.e., <4, 4 to 9, 10 to 15, 16+). Decisions about how best to group demographic data (e.g., grouping the provinces of British Columbia, Alberta, Saskatchewan, and Manitoba together as “Western Canada”) were based on available sample size.

### 2.3. Statistical Analyses

To examine our first two research questions, means were computed for the two occupational stress measures (i.e., organizational, operational), as well as separately for each individual item on those scales. Differences across PSP categories, for both the overall scale scores and for the individual items, were tested using Bonferroni post-hoc tests from a one-way ANOVA model that was calculated first. To examine our third and fourth research questions, multivariate logistic regression models were computed to test the associations between both the overall PSQ-Op and PSQ-Org scores, as well as each of the individual occupational (i.e., specific type of organizational or operational) stressors, and a positive screen for each specific type of mental disorder or the general positive mental disorder screen. The multivariate logistic regression analyses adjusted for sociodemographic covariates (i.e., sex, age, marital status, race/ethnicity, education, urban/rural work location, province of residence, total years of service), the total number of PPTE types (range 0 to 16), and PSP category (i.e., correctional workers and officers, firefighters, paramedics, municipal/provincial police, public safety communications officials, and RCMP). Due to the larger sample size and multiple comparisons in the multivariate logistic regression models, we used a conservative *p*-value of *p* < 0.001. We did not use additional Bonferroni corrections, despite the multiple comparisons, because contemporary theory suggests against such a correction in this type of analytic scenario [62,63].

To assess our fifth research question, a series of nested multivariate logistic regression models were run in order to examine the independent and interactive effects of mean organizational and operational stress scores and the total number of PPTE exposures on each type of positive mental disorder screen and any positive mental disorder screen. The nested multivariate logistic regression models adjusted for sociodemographic covariates and PSP category (Table 2), providing adjusted odds ratios (AORs) describing the relationships between positive screens for each assessed disorder and each of the total number of PPTEs (model 1), the mean organizational stress score (model 2), and the mean operational stress score (model 3). In Table 3, models 1, 2, and 3 serve as baseline assessments of the individual associations between each of the three predictors, separately for each criterion; Model 4 serves as the main analysis, providing the unique associations for each predictor after controlling for the other two predictors, separately for each criterion. Differences in the AORs is a function removing the shared variance between the predictors. Models 4 and 5 examine the statistical interaction between PPTE and each occupational stress measure, separately for each criterion.

### 2.4. Ethics Approval and Consent to Participate

The study was approved by the University of Regina Institutional Research Ethics Board (File #2016-107). We complied with Canadian Psychological Association ethical standards in the treatment of our sample. The survey was available for voluntary participation from 09/01/2016 to 01/31/2017. All interested persons were directed to a website with study details and were required to explicitly indicate consent before proceeding.

## 3. Results

### 3.1. Assessing Organizational and Operational Stressors across PSP Categories

Our first two research questions examined the variability of organizational and operational workplace stressors across PSP categories, as a function of overall scale averages, but also on an item-by-basis. The mean stress levels associated with these occupational stressors, across PSP categories, are provided in Table 1. The two ANOVAs examining the overall mean differences showed significant variability across the various public safety occupational groups, ANOVA *F*(54,394) = 72.21, *p* < 0.001, η^2^ = 0.058 (organizational stressors) and ANOVA *F*(54,380) = 50.95, *p* < 0.001, η^2^ = 0.082 (operational stressors). Across the entire sample, the means for the operational stressor scale items (total mean score 3.17) was significantly lower (*t* = 23.43, *df* = 4440, *p* < 0.01, *r*^2^ = 0.11) than the mean for the organizational stress scale items (total mean score 3.62), which is consistent with prior research [13,28,29,30,31,32].

When we examined differences as a function of each item, we noted many statistically significant effects. Across the entire sample, the specific organizational stressors that were rated the highest were staff shortages (4.46), inconsistent leadership style (4.44), bureaucratic red tape (4.44), a lack of resources (4.29), feeling that different rules apply to different people (4.15), dealing with co-workers (4.05), and feeling like you always have to prove yourself to the organization (4.02). Across all participants, the same organizational stressors tended to be endorsed as having the highest mean level of stress; however, there were some significant differences between specific PSP categories. For example, firefighters tended to report the lowest mean organizational stress scores, whereas RCMP and Correctional Workers tended to report the highest mean organizational stress scores (see Table 1).

Across the entire sample, the operational stressors associated with the highest mean levels of stress were fatigue (4.14), finding time to stay in good physical condition (3.96), occupation-related health issues (3.62), not enough time available to spend with friends and family (3.54), negative comments from the public (3.45), eating healthy at work (3.40), and traumatic events (3.39). Again, the highest rated operational stressors were similar across individual PSP categories, although some significant differences in the actual item means were reported. Specifically, relative to the mean scores of most other operational stressor items, paperwork was associated with higher mean scores for RCMP (4.11) and correctional workers (3.53), while traumatic events were associated with higher mean scores for firefighters (3.48), and shift work was associated with higher mean stress scores for paramedics (3.99) and call center operators/dispatchers (4.01). Overall, across operational stressors, firefighters tended to report the lowest mean stress scores across operational stressors, whereas RCMP tended to report the highest mean stress scores across operational stressors.

### 3.2. Associations between Operational and Organizational Stressors and Mental Health across PSP Categories

The following results address our third research question. The results in Table 2 depict the relationships between organizational and operational stress scores, by an overall scale score and by individual items, and each type of positive mental disorder screen, as well as with any positive mental disorder screens. Again, we intentionally assessed individual items to allow PSP members and leaders to identify potentially actionable opportunities for occupational changes that might benefit PSP mental health. The results are presented as odds ratios after adjustment for sociodemographic covariates, the total number of PPTE exposures, and PSP category. The overall mean scale scores for organizational (adjusted odds ratios (AOR) ranged from 1.34 to 2.08) and operational (adjusted odds ratios (AOR) ranged from 1.41 to 2.30) stress were both associated with increased odds of positive screens for each disorder. Each individual organizational and operational stressor was associated with increased odds of positive screens for PTSD (adjusted odds ratios (AOR) ranged from 1.20 to 1.51), MDD (AORs ranged from 1.13 to 1.62), GAD (AORs ranged from 1.14 to 1.55), SAD (AORs ranged from 1.12 to 1.53), PD (AORs ranged from 1.14 to 1.55), and any mental disorder (AORs ranged from 1.17 to 1.54). There were 8 of 20 organization stressors (significant AORs ranged from 1.15 to 1.34) and 17 of 20 operational stressors (significant AORs ranged from 1.09 to 1.26) associated with increased odds of a positive screen for AUD.

### 3.3. Unique Associations between Organizational Stress, Operational Stress, and Mental Health

The following results address our fourth and fifth research questions, specifically the independent and interactive effects of mean organizational and operational stress total scores on positive mental health screens, after controlling for the number of PPTEs experienced. Our results are provided in Table 3. The results are presented as odds ratios after adjusting for sociodemographic covariates. The Table 3 results provide baseline estimates of the associations between positive screens for each of the assessed mental health disorders, each of the total number of PPTE types (Model 1), the mean organizational stress score (Model 2), and the mean operational stress score (Model 3). With the exception of the relationship between the total number of PPTE types and positive screens for AUD, the total number of PPTE types (AORs ranged from 1.04 to 1.16), the mean organizational stress (AORs ranged from 1.37 to 2.15), and the mean operational stress (AORs ranged from 1.39 to 2.29) scores were all associated with positive screens for each mental disorder category and for the category encompassing any mental disorder when entered into the models independently (i.e., Models 1, 2, and 3). Model 4 examined the unique associations between PPTEs, organizational stressors, operational stressors (i.e., independent variables), and mental health caseness (i.e., dependent variables), with each individual analysis controlling for the other two predictors and the sociodemographic correlates. When the total number of PPTE types and the mean organizational and mean operational stress scores were entered into logistic regression models simultaneously (i.e., Model 4), the total number of PPTE types, the mean organizational stress scores, and the mean operational stress scores remained independently associated with increased odds of positive screens for PTSD (AORs were 1.06, 1.41, and 1.72, respectively), MDD (AORs were 1.04, 1.15, and 1.93, respectively), and PD (AORs were 1.09, 1.24, and 1.76, respectively). In addition, mean organizational and mean operational stress scores were also independently associated with positive screens for GAD (AORs = 1.21 and 1.80, respectively), social anxiety (AORs = 1.14 and 1.80, respectively), and any mental disorder (AORs = 1.24 and 1.96, respectively) (i.e., Model 4). The mean operational stress score was also significantly associated with increased odds of a positive AUD screen (AOR = 1.33). Models 5 and 6 examined whether occupational stress and the number of PPTEs interacted to influence the associations with the mental health variables. Neither of the total number of PPTE types by mean organizational or mean operational stress score interaction terms were significant.

## 4. Discussion

The current results provide evidence that PPTEs are only one element associated with mental health concerns across a wide range of PSP. Operational and organizational stressors associated with doing work in public safety were both significantly associated with all of the mental health variables included in our large survey. Moreover, the associations between both operational and organizational stressors and mental health remained significant, with moderate to large effect sizes, even after the influence of PPTEs was statistically controlled. Accordingly, the current results provide the first large-scale evidence that PSP leaders and managers need to understand the important role of non-traumatic, work-related stressors on the psychological health of their personnel. The current results suggest that organizational and operational workplace stress might even play a larger role on PSP mental health than PPTEs.

The current results can also provide PSP with opportunities to evaluate whether actioning specific individual organizational and operational changes may benefit the mental health of their people. The nature of PSP work (i.e., necessarily high levels of PPTE, life and death decision-making in extreme environments) underscores the need to effectively manage other occupational stressors wherever possible in order to help maximize recruitment, retention, and performance [64]. Participating PSP certainly reported difficulties with being exposed to PPTE types (i.e., a mean score of 11.08 out of 16); however, participants also reported substantial difficulties with most organizational stressors (i.e., mean scores from 2.63 to 4.46 out of 7), such as staff shortages and bureaucratic red tape, which have been underscored as potentially problematic in previous research in other occupations [65,66,67,68]. Substantial difficulties were also reported with most operational stressors (i.e., mean scores from 2.21 to 4.14 out of 7), such as working alone at night and fatigue associated with shift work and over-time. These types of stressors were identified as potentially problematic in previous research conducted in other occupations [69,70,71].

There were significant differences between PSP categories with respect to perceived levels of stress in both the organizational and operational domains. Across organizational stressors overall, RCMP and correctional workers reported the highest levels of stress, whereas firefighters reported the lowest levels. Several significant differences were identified among the individual organizational items that may offer specific directions for leaders looking to make changes. For example, RCMP reported higher levels of stress from a lack of resources when compared to most other PSP, but correctional workers reported higher levels of stress from feeling that different rules apply to different people (e.g., favoritism) than did most other PSP. Among operational stressors, RCMP and paramedics reported the highest levels of overall stress, whereas firefighters reported the lowest levels. Again, there were several significant differences within the operational items that may offer specific directions for leaders looking to make changes. For example, RCMP reported higher levels of stress from trying to find time to stay in good physical condition, but paramedics reported higher levels of stress from fatigue (e.g., shift work, over-time). In any case, the detailed results provide PSP leaders who choose to act with agency to select and test potential solutions they believe might fit best for their individual agency. The differences in organizational and operational stressors may be systemically associated with leadership tools, management structures, workload expectations, environmental variables, or other currently unidentified considerations. The underlying causal factors for each stressor should be identified, where possible, by PSP leadership and then modified, where possible, to minimize mental health impact.

The odds ratios for organizational and operational stressors were comparable; as such, operational stressors are not the only stressors associated with adverse outcomes. The current results suggest that organizational stressors might also offer particularly beneficial intervention targets for PSP managers and senior leaders. Across different positive screenings for mental disorders, the two organizational stressors with the highest odds ratios were believing that feeling like you always have to prove yourself to the organization and believing that if you are sick or injured your co-workers seem to look down on you. The perception that an employee must prove their value to the organization and to other employees may create perceptions of occupational vulnerability and insecurity that could severely negatively impact wellbeing [72,73,74]. Indeed, the current results are consistent with prior evidence that employees’ perceptions of being valued directly impact their workplace orientation, performance, and well-being [75,76,77,78,79]. Assuming the PSP leaders themselves are not already stretched beyond their own capacity, which may be causing compounding problems [80], additional leadership training or support may be helpful to change individual PSP perceptions of being valued by their organization. Across different positive screenings for mental disorders, the two operational stressors with the highest odds ratios were managing social life outside of work and fatigue (e.g., shift work, over-time), both of which might require developing innovative systemic solutions. The PSP categories differed with respect to specific organizational and operational stressors, which suggests individual organizations may also want to consider tailoring efforts to address the specific needs of their members.

The total number of PPTE exposure types was associated with higher odds ratios of screening positive for all measured mental disorders, except alcohol use disorder; in contrast, mean occupational stress scores were associated with higher odds ratios of screening positive for all measured mental disorders. When assessed together, the total number of PPTE exposure types remained significant for PTSD, MDD, and PD, while mean occupational stress scores remained associated with higher odds ratios of screening positive for all measured mental disorders. The interaction terms were not significant, suggesting against a moderating effect. In other words, both PPTE exposure types and occupational stressors appear to be independently contributing to mental disorders, which suggests that if the PPTE cannot be reduced, PSP leadership may still have a significant opportunity to improve mental health by reducing organizational and operational stressors where possible.

### Strengths and Limitations

The current study had several important strengths. First, the results are based on a large and diverse sample that appears demographically representative of the population. Second, the assessment tools have been very broadly psychometrically validated. Third, the statistics used allow for clear interpretations of the relationships of interest. Fourth, the associations of interest were large and statistically significant even using conservative interpretations. Fifth, the results are novel and may directly and indirectly benefit PSP, the research community, and clinical practitioners.

There are also several limitations to the current work that offer directions for future research. First, the PSP sample was self-selected rather than being random and stratified, which means the results may not be broadly representative. Second, responses were anonymous, allowing for potential problems with missing, erroneous, and biased data; furthermore, mental disorder assessments were based on self-report screens instead of diagnoses. In response, future researchers should consider including clinical interviews in order to provide diagnoses. Third, in the results, we used the self-reported number of different PPTE types (which plateaued at 16), rather than exposure frequencies (which plateaued exposure at 11+ times), all of which limits the applicability of results based on retrospective recall and artificial ceilings. In the future, researchers should consider using more accurate and flexible methods for assessing exposure frequency. Fourth, the prevalence and impact of familial stressors and individual difference variables may be significant and substantial, but were not assessed. Thus, there is a need for future researchers to include assessments of the impacts of familial stressors and individual difference variables on PSP mental health. Fifth, an even larger sample size would be required to simultaneously assess the relationships between individual items for each PSP category and each mental disorder. Future researchers might consider tailored data collections within PSP categories for such levels of specificity. Sixth, future researchers may also want to examine whether there are interaction effects between direct and indirect exposures to trauma and each of the individual occupational stressors. Seventh, the current data does not include a comparison group of participants working in other occupations unrelated to public safety; as such, direct comparisons cannot be made to assess relative perceptions of stress between PSP and non-PSP. Future researchers, we suggest, should consider including a non-PSP control group. Eighth, the cross-sectional nature of the data does not allow for potentially important assessments of risk and causality; for example, there is currently no way to know whether traumatic stressors precede and increase vulnerability for occupational stressors, or vice versa, or if the two occur in concert. Longitudinal study designs would help future researchers assess for risk associated with diverse stressors and then identify intervention strategies that can maximally reduce PSP mental health challenges.

## 5. Conclusions

Overall, PSP appear to report significant occupational stress associated with organizational and operational stressors. PPTE exposures may be inevitable for PSP; as such, policy makers should explore ways to mitigate occupational stressors in support of PSP mental health, such as by creating increasingly psychologically safe workplaces. The largest associations appear to be with positive screenings for PTSD and the lowest with positive screenings for AUDs. The current results suggest that a successful action plan to address PTSD and other mental health disorders will likely depend, at least in part, on making changes to reduce organizational and operational stressors within PSP organizations. The largest gains might be made by focusing on leadership training and support, improved organizational engagement, reduced stigma, improved sleep, and strengthening social support.

## Figures and Tables

**Table 1:** Average stress levels associated with occupational stressors across Canadian public safety personnel categories.

Occupational Stressor	Total (*n* = 4441)	Municipal/Provincial Police ^a^ (*n* = 1147)	Federal Police (i.e., RCMP) ^b^ (*n* = 1188)	Corrections ^c^ (*n* = 574)	Firefighters ^d^ (*n* = 711)	Paramedics ^e^ (*n* = 612)	Public Safety Communications Officials [e.g., Call Center Operators/Dispatchers] (*n* = 209)	*F*-Statistic	Significant Differences between PSP Categories
	*M* (*SD*)	*M* (*SD*)	*M* (*SD*)	*M* (*SD*)	*M* (*SD*)	*M* (*SD*)	*M* (*SD*)		
**Organizational Stressors (PSP-Org)**									
**Mean Organizational Stress Score, mean (SD)**	3.62 (1.33)	3.58 (1.30)	3.99 (1.31)	3.93 (1.31)	2.91 (1.22)	3.49 (1.22)	3.57 (1.21)	72.21	a<b, c d<a, b, c, e, f e<b, c f<b, c
Dealing with co-workers	4.05 (1.78)	3.94 (1.74)	4.18 (1.76)	4.65 (1.78)	3.53 (1.75)	4.00 (1.72)	4.25 (1.74)	29.04	a<b, c b<c d<a, b, c, e, f e<c
The feeling that different rules apply to different people (e.g., favouritism)	4.15 (1.95)	4.17 (1.93)	4.22 (1.90)	4.78 (1.86)	3.39 (1.99)	4.11 (1.89)	4.55 (1.87)	36.61	a<c b<c d<a, b, c, e, f e<c
Feeling like you always have to prove yourself to the organization	4.02 (1.96)	3.96 (1.94)	4.37 (1.92)	4.32 (1.95)	3.29 (1.94)	4.03 (1.90)	3.87 (1.92)	31.56	a<b, c d<a, b, c, e, f e<b f<b
Excessive administrative duties	3.69 (1.98)	3.73 (1.87)	4.56 (1.85)	3.98 (1.98)	2.75 (1.81)	2.95 (1.84)	2.99 (1.82)	116.64	a<b c<b d<a, b e<a, b, c f<a, c
Constant change in policy/legislation	3.80 (1.91)	3.63 (1.84)	4.04 (1.85)	4.40 (1.93)	3.08 (1.80)	3.80 (1.93)	4.22 (1.92)	41.19	a<b, c, f b<c d<a, b, c, e, f e<c
Staff shortages	4.46 (2.08)	4.48 (1.96)	5.19 (1.86)	4.51 (2.03)	3.07 (1.96)	4.30 (2.10)	5.32 (1.86)	113.31	a<b, f c<b, f d<a, b, c, e, f e<b, f
Bureaucratic red tape	4.44 (1.98)	4.35 (1.91)	4.83 (1.87)	4.91 (1.89)	3.60 (1.99)	4.37 (2.07)	4.41 (2.03)	43.40	a<b, c d<a, b, c, e, f e<b, c f<c
Too much computer work	3.19 (1.89)	3.26 (1.83)	3.99 (1.88)	3.32 (1.92)	2.45 (1.65)	2.47 (1.62)	2.58 (1.70)	96.29	a<b c<b d<a, b, c e<a, b, c f<a, b, c
Lack of training on new equipment	3.12 (1.80)	2.82 (1.69)	3.41 (1.82)	3.38 (1.97)	3.09 (1.78)	2.96 (1.74)	2.97 (1.74)	16.68	a<b, c, d d<b e<b, c f<c
Perceived pressure to volunteer free time	2.64 (1.85)	2.51 (1.77)	3.21 (2.03)	2.34 (1.76)	2.55 (1.74)	2.39 (1.71)	1.95 (1.43)	37.08	a<b c<b d<b e<b f<a, b, d, e
Dealing with supervisors	3.69 (2.00)	3.53 (1.96)	3.92 (1.97)	4.20 (2.02)	3.25 (1.96)	3.63 (2.01)	3.55 (1.93)	19.75	a<b, c d<a, b, c, e e<b, c f<c
Inconsistent leadership style	4.44 (2.10)	4.29 (2.09)	4.56 (2.05)	4.97 (2.09)	3.87 (2.12)	4.65 (2.06)	4.55 (2.06)	21.48	a<b, c, e b<c d<a, b, c, e, f
Lack of resources	4.29 (2.04)	4.05 (2.00)	4.95 (1.91)	4.55 (1.99)	3.26 (1.89)	4.46 (2.05)	4.10 (2.03)	72.27	a<b, c, e c<b d<a, b, c, e, f e<b f<b
Unequal sharing of work responsibilities	3.90 (2.10)	3.81 (2.06)	4.18 (2.06)	4.57 (2.06)	3.00 (1.92)	3.93 (2.10)	4.02 (2.08)	45.16	a<b, c b<c d<a, b, c, e, f e<c f<c
If you are sick or injured your co-workers seem to look down on you	2.78 (2.00)	2.80 (2.01)	3.19 (2.13)	2.91 (1.99)	2.00 (1.56)	2.59 (1.96)	3.12 (1.98)	35.63	a<b d<a, b, c, e, f e<b, f
Leaders over-emphasize the negatives (e.g., supervisor evaluations, public complaints)	3.51 (2.10)	3.43 (2.06)	3.61 (2.10)	3.81 (2.16)	2.90 (1.91)	3.82 (2.16)	3.84 (2.15)	19.32	a<c, e d<a, b, c, e, f
Internal investigations	3.04 (2.08)	3.22 (2.14)	3.06 (2.11)	3.59 (2.19)	2.24 (1.63)	3.17 (2.07)	2.82 (1.92)	32.87	a<c b<c d<a, b, c, e, f e<c f<c
Dealing with the court system	2.63 (1.84)	3.40 (1.88)	3.28 (1.81)	2.24 (1.65)	1.52 (1.25)	1.95 (1.52)	1.64 (1.28)	188.89	c<a, b d<a, b, c, e e<a, b, c f<a, b, c
The need to be accountable for doing your job	3.35 (1.92)	3.48 (1.99)	3.46 (1.87)	3.59 (2.00)	2.82 (1.77)	3.30 (1.88)	3.34 (1.97)	14.46	d<a, b, c, e, f
Inadequate equipment	3.13 (1.89)	2.86 (1.76)	3.59 (1.93)	3.45 (2.00)	2.55 (1.63)	3.11 (1.91)	3.23 (1.93)	37.02	a<b, c d<a, b, c, e, f e<b, c
**Operational Stressors (PSP-Op)**									
**Mean Operational Stress Score, *M* (*SD*)**	3.17 (1.28)	3.15 (1.29)	3.54 (1.36)	3.06 (1.22)	2.62 (1.09)	3.30 (1.20)	2.96 (1.07)	50.95	a<b c<b, e d<a, b, e, f e<b f<b, c, e
Shift work	3.32 (2.10)	3.38 (2.09)	3.29 (2.15)	2.76 (2.18)	2.96 (1.82)	3.99 (2.04)	4.01 (2.00)	30.49	a<e, f b<e, f c<a, b, e, f d<a, b, e, f
Working alone at night	2.21 (1.86)	2.39 (1.85)	2.91 (2.20)	1.90 (1.68)	1.44 (1.10)	1.94 (1.69)	1.56 (1.13)	76.55	a<b c<a, b d<a, b, c, e e<a, b f<a, b
Over-time demands	2.68 (1.94)	2.70 (1.86)	3.29 (2.08)	2.08 (1.67)	1.75 (1.38)	3.14 (2.09)	2.54 (1.77)	80.24	a<b, e c<a, b, e, f d<a, b, c, e, f f<b, e
Risk of being injured on the job	2.98 (1.89)	2.69 (1.76)	3.16 (1.98)	3.29 (1.94)	2.86 (1.73)	3.55 (1.95)	1.49 (1.09)	50.95 ***	a<b, c, e b<e d<b, c, e f<a, b, c, d, e
Work-related activities on days off (e.g., court, community events)	2.53 (1.77)	2.87 (1.82)	3.16 (1.92)	1.92 (1.43)	1.96 (1.41)	2.24 (1.63)	1.69 (1.21)	86.41	a<b c<a, b, e d<a, b, e e<a, b f<a, b, e
Potentially psychologically traumatic events (e.g., motor vehicle accidents, domestics, death, injury)	3.39 (1.98)	3.21 (1.94)	3.64 (2.10)	2.80 (1.95)	3.48 (1.82)	3.73 (1.89)	3.38 (1.93)	20.20	a<b, e c<a, b, d, e, f
Managing your social life outside of work	3.02 (1.77)	2.88 (1.69)	3.23 (1.88)	2.97 (1.75)	2.59 (1.60)	3.28 (1.81)	3.38 (1.72)	18.00	a<b, e, f c<b, e d<a, b, c, e, f
Not enough time available to spend with friends and family	3.54 (1.87)	3.44 (1.81)	3.83 (1.96)	3.45 (1.82)	2.94 (1.73)	3.86 (1.85)	3.91 (1.76)	27.20	a<b, e, f c<b, e, f d<a, b, c, e, f
Paperwork	3.29 (1.88)	3.38 (1.81)	4.11 (1.83)	3.53 (1.89)	2.36 (1.59)	2.84 (1.67)	1.96 (1.30)	126.64	a<b c<b d<a, b, c, e e<a, b, c f<a, b, c, e
Eating healthy at work	3.40 (1.84)	3.38 (1.85)	3.76 (1.78)	3.32 (1.87)	2.50 (1.59)	3.79 (1.82)	3.52 (1.85)	51.38	a<b, e c<b, e d<a, b, c, e, f
Finding time to stay in good physical condition	3.96 (1.85)	3.87 (1.85)	4.32 (1.78)	4.03 (1.82)	3.21 (1.81)	4.18 (1.79)	4.01 (1.78)	36.73	a<b, e c<b d<a, b, c, e, f
Fatigue (e.g., shift work, over-time)	4.14 (1.99)	4.07 (1.97)	4.27 (1.99)	3.91 (2.11)	3.62 (1.88)	4.66 (1.88)	4.70 (1.88)	24.81	a<e, f b<f c<b, e, f d<a, b, e, f
Occupation-related health issues (e.g., back pain)	3.62 (2.02)	3.55 (2.01)	3.94 (1.96)	3.72 (2.08)	3.07 (1.90)	3.70 (2.07)	3.49 (2.01)	17.76	a d<a, b, c, e f<b
Lack of understanding from family and friends about your work	3.04 (1.89)	2.88 (1.83)	3.10 (1.91)	3.39 (2.00)	2.74 (1.79)	3.19 (1.92)	3.18 (1.86)	10.40	a<c, e b<c d<b, c, e, f
Making friends outside the job	2.73 (1.84)	2.43 (1.69)	3.12 (1.91)	2.90 (1.97)	2.20 (1.59)	2.94 (1.90)	2.83 (1.86)	32.01	a<b, c, e, f d<b, c, e, f
Upholding a “higher image” in public	2.98 (1.89)	3.07 (1.95)	3.36 (1.91)	2.68 (1.79)	2.58 (1.79)	2.97 (1.81)	2.52 (1.67)	22.53	a<b c<a, b d<a, b, e e<b f<a, b, e
Negative comments from the public	3.45 (1.97)	3.83 (2.00)	3.80 (1.93)	3.25 (1.96)	2.80 (1.82)	3.12 (1.89)	3.08 (1.84)	39.31	c<a, b d<a, b, c, e e<a, b f<a, b
Limitations to your social life (e.g., who your friends are, where you socialize)	2.99 (1.87)	2.92 (1.86)	3.35 (1.92)	3.05 (1.91)	2.40 (1.64)	3.05 (1.88)	2.95 (1.81)	23.85	a<b c<b d<a, b, c, e, f e<b
Feeling like you are always on the job	3.31 (2.01)	3.37 (1.96)	3.86 (2.01)	3.07 (1.98)	2.74 (1.93)	3.31 (1.98)	2.49 (1.69)	39.84	a<b c<a, b d<a, b, c, e e<b f<a, b, c, e
Friends/family feel the effects of the stigma associated with your job	2.87 (1.88)	2.99 (1.86)	3.31 (1.94)	2.87 (1.94)	2.28 (1.68)	2.65 (1.77)	2.45 (1.68)	32.66	a<b c<b d<a, b, e e<a, b, c f<a, b

Notes. PSP = Public Safety Personnel. Different lettered superscripts indicate PSP categories that differ from one another at *p* ≤ 0.05. Differences in mean scores across PSP categories were tested using Bonferroni post-hoc tests from the one-way ANOVA models. *** all *F*-values were statistically significant at *p* ≤ 0.001.

**Table 2:** Relationship between occupational stressors and positive mental disorder screens among Canadian public safety personnel.

Occupational Stressor	PTSD	MDD	Generalized Anxiety	Social Anxiety	Panic Disorder	Alcohol Use Disorder	Any Mental Disorder
	AOR (95% CI)	AOR (95% CI)	AOR (95% CI)	AOR (95% CI)	AOR (95% CI)	AOR (95% CI)	AOR (95% CI)
**Organizational Stressors (PSP-Org)**							
**Mean Organizational Stress Score**	2.08 *** (1.92, 2.25)	1.82 *** (1.69, 1.95)	1.85 *** (1.71, 2.00)	1.74 *** (1.60, 1.90)	1.88 *** (1.69, 2.10)	1.34 *** (1.19, 1.50)	1.94 *** (1.81, 2.08)
Dealing with co-workers	1.35 *** (1.28, 1.42)	1.35 *** (1.28, 1.41)	1.40 *** (1.33, 1.48)	1.34 *** (1.26, 1.42)	1.33 *** (1.24, 1.43)	1.19 *** (1.09, 1.29)	1.38 *** (1.32, 1.44)
The feeling that different rules apply to different people (e.g., favoritism)	1.41 *** (1.34, 1.48)	1.33 *** (1.27, 1.39)	1.35 *** (1.29, 1.42)	1.34 *** (1.27, 1.42)	1.35 *** (1.26, 1.45)	1.15 *** (1.07, 1.24)	1.38 *** (1.32, 1.44)
Feeling like you always have to prove yourself to the organization	1.51 *** (1.44, 1.59)	1.43 *** (1.37, 1.50)	1.50 *** (1.43, 1.59)	1.42 *** (1.34, 1.50)	1.41 *** (1.31, 1.52)	1.21 *** (1.12, 1.30)	1.48 *** (1.41, 1.54)
Excessive administrative duties	1.30 *** (1.24, 1.37)	1.24 *** (1.19, 1.30)	1.24 *** (1.18, 1.30)	1.22 *** (1.16, 1.28)	1.25 *** (1.17, 1.33)	1.08 (0.999, 1.16)	1.26 *** (1.20, 1.31)
Constant change in policy/legislation	1.33 *** (1.27, 1.40)	1.33 *** (1.27, 1.39)	1.31 *** (1.25, 1.38)	1.28 *** (1.21, 1.34)	1.25 *** (1.16, 1.33)	1.17 *** (1.08, 1.27)	1.33 *** (1.28, 1.39)
Staff shortages	1.27 *** (1.21, 1.33)	1.23 *** (1.17, 1.28)	1.24 *** (1.18, 1.31)	1.21 *** (1.15, 1.28)	1.24 *** (1.16, 1.33)	1.02 (0.94, 1.10)	1.22 *** (1.17, 1.27)
Bureaucratic red tape	1.34 *** (1.28, 1.41)	1.28 *** (1.22, 1.34)	1.26 *** (1.20, 1.33)	1.28 *** (1.21, 1.35)	1.26 *** (1.17, 1.36)	1.13 (1.04, 1.22)	1.30 *** (1.24, 1.35)
Too much computer work	1.21 *** (1.16, 1.27)	1.19 *** (1.14, 1.24)	1.19 *** (1.14, 1.25)	1.12 *** (1.06, 1.18)	1.17 *** (1.10, 1.25)	1.11 (1.02, 1.20)	1.20 *** (1.15, 1.25)
Lack of training on new equipment	1.26 *** (1.21, 1.32)	1.23 *** (1.18, 1.29)	1.24 *** (1.18, 1.30)	1.27 *** (1.20, 1.33)	1.24 *** (1.16, 1.32)	1.09 (1.01, 1.17)	1.27 *** (1.22, 1.33)
Perceived pressure to volunteer free time	1.28 *** (1.22, 1.34)	1.21 *** (1.16, 1.26)	1.22 *** (1.17, 1.28)	1.17 *** (1.11, 1.22)	1.24 *** (1.16, 1.31)	1.09 (1.02, 1.18)	1.25 *** (1.20, 1.30)
Dealing with supervisors	1.37 *** (1.31, 1.44)	1.30 *** (1.25, 1.35)	1.32 *** (1.26, 1.38)	1.24 *** (1.19, 1.31)	1.32 *** (1.24, 1.41)	1.16 *** (1.08, 1.25)	1.35 *** (1.30, 1.40)
Inconsistent leadership style	1.42 *** (1.35, 1.49)	1.29 *** (1.24, 1.35)	1.30 *** (1.24, 1.36)	1.22 *** (1.16, 1.28)	1.32 *** (1.23, 1.42)	1.08 (1.01, 1.16)	1.32 *** (1.27, 1.38)
Lack of resources	1.35 *** (1.29, 1.42)	1.25 *** (1.19, 1.30)	1.25 *** (1.19, 1.32)	1.28 *** (1.21, 1.35)	1.28 *** (1.19, 1.37)	1.10 (1.02, 1.19	1.28 *** (1.23, 1.33)
Unequal sharing of work responsibilities	1.35 *** (1.29, 1.41)	1.27 *** (1.22, 1.32)	1.24 *** (1.19, 1.30)	1.27 *** (1.21, 1.33)	1.31 *** (1.23, 1.39)	1.09 (1.02, 1.18)	1.30 *** (1.25, 1.35)
If you are sick or injured your co-workers seem to look down on you	1.51 *** (1.44, 1.57)	1.44 *** (1.38, 1.50)	1.47 *** (1.40, 1.53)	1.35 *** (1.29, 1.41)	1.55 *** (1.46, 1.65)	1.20 *** (1.12, 1.29)	1.53 *** (1.46, 1.59)
Leaders over-emphasize the negatives (e.g., supervisor evaluations, public complaints)	1.37 *** (1.32, 1.43)	1.28 *** (1.23, 1.33)	1.29 *** (1.23, 1.34)	1.25 *** (1.19, 1.31)	1.31 *** (1.23, 1.39)	1.15 *** (1.07, 1.23)	1.32 *** (1.27, 1.37)
Internal investigations	1.28 *** (1.23, 1.33)	1.21 *** (1.16, 1.26)	1.21 *** (1.16, 1.27)	1.18 *** (1.13, 1.24)	1.25 *** (1.18, 1.32)	1.15 *** (1.07, 1.23)	1.24 *** (1.20, 1.29)
Dealing with the court system	1.21 *** (1.15, 1.27)	1.13 *** (1.08, 1.19)	1.16 *** (1.10, 1.22)	1.19 *** (1.12, 1.25)	1.21 *** (1.13, 1.29)	1.19 *** (1.09, 1.29)	1.17 *** (1.12, 1.22)
The need to be accountable for doing your job	1.26 *** (1.21, 1.32)	1.27 *** (1.22, 1.32)	1.28 *** (1.22, 1.34)	1.27 *** (1.21, 1.33)	1.26 *** (1.18, 1.34)	1.23 *** (1.15, 1.33)	1.32 *** (1.27, 1.38)
Inadequate equipment	1.28 *** (1.22, 1.34)	1.22 *** (1.17, 1.28)	1.20 *** (1.14, 1.25)	1.23 *** (1.17, 1.29)	1.25 *** (1.17, 1.33)	1.08 (0.999, 1.16)	1.25 *** (1.19, 1.30)
**Operational Stressors (PSP-Op)**							
**Mean Operational Stress Score**	2.19 *** (2.02, 2.38)	2.14 *** (1.98, 2.31)	2.07 *** (1.91, 2.25)	1.98 *** (1.82, 2.16)	2.05 *** (1.83, 2.28)	1.41 *** (1.25, 1.58)	2.30 *** (2.13, 2.48)
Shift work	1.20 *** (1.15, 1.25)	1.19 *** (1.15, 1.24)	1.14 *** (1.10, 1.19)	1.16 *** (1.11, 1.22)	1.14 *** (1.07, 1.20)	1.09 (1.02, 1.17)	1.21 *** (1.16, 1.25)
Working alone at night	1.24 *** (1.19, 1.30)	1.20 *** (1.15, 1.26)	1.19 *** (1.13, 1.24)	1.21 *** (1.15, 1.27)	1.24 *** (1.16, 1.31)	1.02 (0.94, 1.11)	1.20 *** (1.15, 1.25)
Over-time demands	1.22 *** (1.16, 1.27)	1.23 *** (1.18, 1.28)	1.21 *** (1.15, 1.26)	1.19 *** (1.14, 1.25)	1.25 *** (1.17, 1.33)	1.07 (0.99, 1.15)	1.23 *** (1.18, 1.28)
Risk of being injured on the job	1.34 *** (1.28, 1.50)	1.28 *** (1.22, 1.33)	1.29 *** (1.23, 1.35)	1.28 *** (1.22, 1.35)	1.38 *** (1.30, 1.47)	1.13 *** (1.05, 1.22)	1.31 *** (1.26, 1.37)
Work-related activities on days off (e.g., court, community events)	1.32 *** (1.26, 1.39)	1.25 *** (1.19, 1.31)	1.25 *** (1.19, 1.31)	1.23 *** (1.17, 1.30)	1.27 *** (1.19, 1.36)	1.07 (0.98, 1.16)	1.28 *** (1.22, 1.34)
Potentially psychologically traumatic events (e.g., motor vehicle accidents, domestics, death, injury)	1.48 *** (1.41, 1.55)	1.37 *** (1.31, 1.43)	1.39 *** (1.32, 1.46)	1.32 *** (1.26, 1.39)	1.45 *** (1.35, 1.54)	1.26 *** (1.17, 1.36)	1.43 *** (1.37, 1.49)
Managing your social life outside of work	1.46 *** (1.39, 1.53)	1.56 *** (1.49, 1.64)	1.55 *** (1.47, 1.63)	1.47 *** (1.39, 1.56)	1.49 *** (1.39, 1.59)	1.26 *** (1.16, 1.36)	1.58 *** (1.50, 1.66)
Not enough time available to spend with friends and family	1.37 *** (1.30, 1.44)	1.34 *** (1.28, 1.41)	1.38 *** (1.31, 1.46)	1.26 *** (1.20, 1.33)	1.32 *** (1.23, 1.41)	1.11 (1.02, 1.20)	1.35 *** (1.29, 1.41)
Paperwork	1.26 *** (1.20, 1.32)	1.24 *** (1.19, 1.30)	1.25 *** (1.19, 1.31)	1.20 *** (1.13, 1.26)	1.27 *** (1.18, 1.36)	1.16 *** (1.07, 1.26)	1.25 *** (1.20, 1.31)
Eating healthy at work	1.34 *** (1.28, 1.41)	1.40 *** (1.34, 1.47)	1.33 *** (1.27, 1.40)	1.31 *** (1.24, 1.38)	1.29 *** (1.21, 1.38)	1.18 *** (1.09, 1.27)	1.41 *** (1.35, 1.48)
Finding time to stay in good physical condition	1.39 *** (1.32, 1.46)	1.50 *** (1.43, 1.57)	1.42 *** (1.34, 1.50)	1.33 *** (1.26, 1.41)	1.37 *** (1.27, 1.48)	1.19 *** (1.09, 1.29)	1.49 *** (1.42, 1.56)
Fatigue (e.g., shift work, over-time)	1.47 *** (1.40, 1.55)	1.62 *** (1.54, 1.71)	1.48 *** (1.40, 1.56)	1.39 *** (1.31, 1.47)	1.51 *** (1.40, 1.63)	1.22 *** (1.13, 1.32)	1.54 *** (1.47, 1.61)
Occupation-related health issues (e.g., back pain)	1.41 *** (1.35, 1.47)	1.40 *** (1.34, 1.46)	1.38 *** (1.32, 1.45)	1.30 *** (1.24, 1.37)	1.45 *** (1.35, 1.55)	1.17 *** (1.09, 1.26)	1.38 *** (1.33, 1.44)
Lack of understanding from family and friends about your work	1.45 *** (1.39, 1.52)	1.37 *** (1.32, 1.43)	1.38 *** (1.32, 1.45)	1.37 *** (1.30, 1.44)	1.38 *** (1.30, 1.47)	1.23 *** (1.15, 1.33)	1.46 *** (1.40, 1.53)
Making friends outside the job	1.38 *** (1.32, 1.45)	1.40 *** (1.34, 1.47)	1.35 *** (1.28, 1.41)	1.53 *** (1.45, 1.61)	1.36 *** (1.28, 1.45)	1.15 *** (1.07, 1.24)	1.44 *** (1.38, 1.51)
Upholding a “higher image” in public	1.38 *** (1.32, 1.45)	1.38 *** (1.32, 1.44)	1.41 *** (1.34, 1.48)	1.39 *** (1.33, 1.47)	1.33 *** (1.25, 1.42)	1.23 *** (1.14, 1.33)	1.43 *** (1.37, 1.50)
Negative comments from the public	1.39 *** (1.32, 1.45)	1.32 *** (1.26, 1.38)	1.39 *** (1.32, 1.46)	1.38 *** (1.31, 1.45)	1.31 *** (1.23, 1.40)	1.15 *** (1.06, 1.23)	1.41 *** (1.35, 1.47)
Limitations to your social life (e.g., who your friends are, where you socialize)	1.40 *** (1.34, 1.47)	1.41 *** (1.35, 1.47)	1.39 *** (1.32, 1.46)	1.41 *** (1.34, 1.48)	1.34 *** (1.26, 1.43)	1.20 *** (1.11, 1.29)	1.45 *** (1.39, 1.51)
Feeling like you are always on the job	1.47 *** (1.40, 1.54)	1.42 *** (1.36, 1.49)	1.44 *** (1.37, 1.51)	1.37 *** (1.30, 1.44)	1.42 *** (1.33, 1.51)	1.14 *** (1.06, 1.23)	1.45 *** (1.39, 1.51)
Friends/family feel the effects of the stigma associated with your job	1.49 *** (1.42, 1.56)	1.39 *** (1.33, 1.45)	1.46 *** (1.39, 1.54)	1.41 *** (1.34, 1.48)	1.46 *** (1.37, 1.56)	1.25 *** (1.16, 1.34)	1.48 *** (1.41, 1.55)

Notes. PTSD = post-traumatic stress disorder; MDD = major depressive disorder; AOR = Odds ratio adjusted for sex, age, marital status, race/ethnicity, education, urban/rural work location, province of residence, the total years of service, the total number of trauma exposures, and the public safety officer category. *** *p* < 0.001.

**Table 3:** Relationship between potentially psychologically traumatic event exposures, organizational stressors, and operational stressors, and any positive mental disorder screen among Canadian public safety personnel.

	PTSD	MDD	Generalized Anxiety	Social Anxiety	Panic Disorder	Alcohol Use Disorder	Any Mental Disorder
	AOR (95% CI)	AOR (95% CI)	AOR (95% CI)	AOR (95% CI)	AOR (95% CI)	AOR (95% CI)	AOR (95% CI)
**Model 1**							
Total Number of Potentially Psychologically Traumatic Event Exposure Types	1.13 *** (1.09, 1.16)	1.10 *** (1.07, 1.13)	1.08 *** (1.05, 1.11)	1.04 (1.01, 1.07)	1.16 *** (1.10, 1.21)	1.05 (0.996, 1.10)	1.07 (1.05, 1.10)
**Model 2**							
Mean Organizational Stress Score	2.15 *** (2.00, 2.31)	1.90 *** (1.78, 2.03)	1.90 *** (1.77, 2.05)	1.75 *** (1.62, 1.89)	1.96 *** (1.77, 2.16)	1.37 *** (1.24, 1.53)	1.99 *** (1.87, 2.12)
**Model 3**							
Mean Operational Stress Score	2.28 *** (2.12, 2.46)	2.18 *** (2.04, 2.34)	2.09 *** (1.94, 2.25)	1.98 *** (1.80, 2.09)	2.09 *** (1.89, 2.31)	1.39 *** (1.25, 1.55)	2.29 *** (2.14, 2.46)
**Model 4**							
Total Number of Potentially Psychologically Traumatic Event Exposure Types	1.06 *** (1.03, 1.10)	1.04 (1.01, 1.07)	1.01 (0.98, 1.04)	0.97 (0.94, 1.00)	1.09 *** (1.04, 1.15)	1.01 (0.96, 1.06)	1.01 (0.98, 1.04)
Mean Organizational Stress Score	1.41 *** (1.26, 1.58)	1.15 (1.04, 1.28)	1.21 *** (1.08, 1.36)	1.14 (1.01, 1.28)	1.24 (1.05, 1.45)	1.07 (0.89, 1.50)	1.24 *** (1.12, 1.37)
Mean Operational Stress Score	1.72 *** (1.54, 1.92)	1.93 *** (1.73, 2.14)	1.80 *** (1.61, 2.03)	1.80 *** (1.59, 2.03)	1.76 *** (1.51, 2.06)	1.33 (1.11, 1.59)	1.96 *** (1.76, 2.17)
**Model 5**							
Trauma Exposure by Organizational Stress Interaction Term	1.01 (0.98, 1.04)	1.02 (0.99, 1.04)	1.01 (0.98, 1.03)	1.02 (0.99, 1.04)	1.00 (0.96, 1.04)	0.98 (0.95, 1.02)	1.00 (0.98, 1.02)
**Model 6**							
Trauma Exposure by Operational Stress Interaction Term	1.00 (0.98, 1.03)	1.01 (0.98, 1.03)	1.00 (0.98, 1.03)	1.01 (0.98, 1.03)	0.99 (0.95, 1.03)	0.98 (0.94, 1.01)	0.99 (0.97, 1.02)

Notes. AOR = Odds ratio adjusted for sociodemographic covariates (i.e., sex, age, marital status, race/ethnicity, education, urban/rural work location, province of residence, total years of service, public safety category); PTSD = post-traumatic stress disorder; MDD = major depressive disorder. Model 1: The total number of potentially psychologically traumatic event types entered and adjusted for covariates. Model 2: The mean organizational stress score entered and adjusted for covariates. Model 3: The mean operational stress score entered and adjusted for SD covariates. Model 4: The total number of potentially psychologically traumatic event types, the mean organizational stress score, and the mean operational stress score entered into the same model simultaneously and adjusted for covariates. Model 5: Model 4 with the main effects of the total number of potentially psychologically traumatic event types, the mean organizational stress score, and the interaction term for the total number of potentially psychologically traumatic event types x the mean organizational stress score and adjusted for covariates and the mean operational stress score. Model 6: Model 4 with the main effects of the total number of potentially psychologically traumatic event types and the mean operational stress score and the interaction term for the total number of potentially psychologically traumatic event types x the mean operational stress score and adjusted for covariates and the mean organizational stress score. *** *p* < 0.001.
